# Alkaloids from the Sponge *Stylissa carteri* Present Prospective Scaffolds for the Inhibition of Human Immunodeficiency Virus 1 (HIV-1)

**DOI:** 10.3390/md14020028

**Published:** 2016-02-04

**Authors:** Aubrie O’Rourke, Stephan Kremb, Theresa Maria Bader, Markus Helfer, Philippe Schmitt-Kopplin, William H. Gerwick, Ruth Brack-Werner, Christian R. Voolstra

**Affiliations:** 1 Red Sea Research Center, Division of Biological and Environmental Science and Engineering, King Abdullah University of Science and Technology (KAUST), Thuwal 23955-6900, Saudi Arabia; aubrie.orourke@kaust.edu.sa (A.O.); stephangeorg.kremb@kaust.edu.sa (S.K.); 2 Research Unit Analytical BioGeoChemistry, Helmholtz Zentrum Muenchen, 85764 Neuherberg, Germany; theresa.bader@helmholtz-muenchen.de (T.M.B.); schmitt-kopplin@helmholtz-muenchen.de (P.S.-K.); 3 Institute of Virology, Helmholtz Zentrum Muenchen, German Research Center for Environmental Health, Ingolstädter Landstraße 1, D-85764 Neuherberg, Germany; markus.helfer@helmholtz-muenchen.de (M.H.); brack@helmholtz-muenchen.de (R.B.W.); 4 Analytical Food Chemistry, Technical University of Munich, 85354 Freising Weihenstephan, Germany; 5 Scripps Institution of Oceanography and Skaggs School of Pharmacy and Pharmaceutical Sciences, University of California San Diego, 9500 Gilman Drive, La Jolla, CA 92093, USA; wgerwick@ucsd.edu

**Keywords:** *Stylissa carteri*, marine bioprospecting, HIV-1, reverse transcriptase, Red Sea

## Abstract

The sponge *Stylissa carteri* is known to produce a number of secondary metabolites displaying anti-fouling, anti-inflammatory, and anti-cancer activity. However, the anti-viral potential of metabolites produced by *S. carteri* has not been extensively explored. In this study, an *S. carteri* extract was HPLC fractionated and a cell based assay was used to evaluate the effects of HPLC fractions on parameters of Human Immunodeficiency Virus (HIV-1) infection and cell viability. Candidate HIV-1 inhibitory fractions were then analyzed for the presence of potential HIV-1 inhibitory compounds by mass spectrometry, leading to the identification of three previously characterized compounds, *i.e.*, debromohymenialdisine (DBH), hymenialdisine (HD), and oroidin. Commercially available purified versions of these molecules were re-tested to assess their antiviral potential in greater detail. Specifically, DBH and HD exhibit a 30%–40% inhibition of HIV-1 at 3.1 μM and 13 μM, respectively; however, both exhibited cytotoxicity. Conversely, oroidin displayed a 50% inhibition of viral replication at 50 μM with no associated toxicity. Additional experimentation using a biochemical assay revealed that oroidin inhibited the activity of the HIV-1 Reverse Transcriptase up to 90% at 25 μM. Taken together, the chemical search space was narrowed and previously isolated compounds with an unexplored anti-viral potential were found. Our results support exploration of marine natural products for anti-viral drug discovery.

## 1. Introduction

In 2013, there were an estimated 35.3 million people infected and living with HIV [1], and the number has continued to increase. Drugs currently used as anti-HIV-1 therapeutics fall under five categories, including inhibitors of viral entry, membrane fusion, the reverse transcriptase, the integrase, and the protease. Highly active anti-retroviral therapy (HAART) combines drugs from at least two of the different classes of the anti-retroviral agents available [2]. Nevertheless, the emergence of drug resistant retroviruses is still a major issue associated with current anti-HIV-1 drug therapies [3]. Hence, the effectiveness of HIV therapy relies on the discovery and approval of novel therapeutics that are able to combat the various viral replication phases of HIV.

The first anti-retroviral medicine, azidothymidine (AZT), was approved in 1987 for the treatment of HIV-1 [4]. This drug terminates DNA strand elongation by the addition of an azide group at the C-3 of the nucleoside sugar. This modification to the 5-carbon sugar is thought to be inspired by the nucleoside analogs produced by the sponge *Tethya crypta,* which possess an arabinose sugar instead of the deoxyribose sugar required for DNA strand elongation [5]. By substituting the groups bound to the 2′ and 3′ carbons of the nucleoside sugar, a number of HIV-1 reverse transcriptase inhibitors were generated. However, in the past 15 years of clinical trials only five novel natural product pharmacophores were investigated in clinical trials. The majority of the 133 naturally derived compounds in clinical trials for the period of 2008–2013 are derivatives of existing pharmacophores that are already present in existing human medicines. For this same time period, only 2 of the 133 compounds, both derivatives of the cyclosporin A pharmacophore, were investigated as anti-viral therapeutic candidates in the treatment of the Hepatitis C Virus (HCV) [6]. This reveals that there is a need for novel anti-viral pharmacophores and their derivatives in clinical trials.

A statistical review by Hu *et al.* [7] illustrates that bioactivities were assigned to only approximately 25% of the marine natural products reported in the literature from 1985 to 2012. This does not mean that the remaining 75% do not possess bioactivity; instead, it suggests a discrepancy between the rate of discovery for marine natural products and the investigation of their associated bioactivities. A further look into the type of bioactivities reported revealed that 56% of the bioactive compounds were associated with anti-cancer activity but only 3% with anti-viral activity. Interestingly, in years where a greater variety of disease targets were screened in order to identify inhibitors, the proportion of reported bioactivity was the greatest. This illustrates that there is a need to expand marine natural product screening efforts beyond the detection of anti-cancer activity in order to fully realize the potential utility of marine secondary metabolites.

In this study, we investigated the anti-viral potential of *Stylissa carteri*, in the family Scopalinidae, collected from the Red Sea. *S. carteri* is known to produce a number of pharmacologically active brominated pyrrole-2-aminoimidazole alkaloids, which are also produced by sponges from the families Agelasidae, Axindellidae, Hymeniacidonidae [8]. A review of the literature shows that the chemical repertoire of *Stylissa* sp. is well characterized with nearly 100 compounds reported. Among these compounds, a dimer of oroidin known as sceptrin along with its derivatives debromosceptrin, dibromosceptrin, and oxysceptrin have been reported to inhibit Herpes Simplex Virus-1 (HSV-1) and Vesicular Stomatitis Virus (VSV) [9]. This lends support to our aim of identifying molecules with anti-viral activity from *S. carteri*. More specific to anti-retroviral activity, another derivative of oroidin, hymenialdisine (HD), was used experimentally to inhibit Nuclear Factor-kB (NFkB), and subsequently inhibit transcription from the long terminal repeat (LTR) of the Human Immunodeficiency Virus (HIV) *in vitro* [10]*.* Here, we tested 11 high-performance liquid chromatography (HPLC) fractions obtained from *S. carteri* on a well-established cell-based screening system (EASY-HIT, [11]), which evaluates both anti-HIV activity and cytotoxicity in order to identify compounds with anti-HIV bioactivity from the Red Sea sponge *S. carteri.*

## 2. Results and Discussion

### 2.1. Biological Activities of Fractions of S. carteri Extracts

*S. carteri* specimens collected from different coral reefs in the Red Sea were subjected to Solid Phase Extraction (SPE) under different conditions to generate three desalted fractions enriched for pharmacologically relevant small organic compounds of moderate polarity. These SPE fractions were subsequently evaluated for biological activity with an assay system (EASY-HIT; [11]) that co-monitors parameters of HIV-infection (*i.e.*, expression of an HIV-dependent reporter gene) and cell viability (*i.e.*, metabolic activity of cells). This assay system has been validated for HIV-inhibitor screening and has been successfully used in the past to evaluate anti-HIV activity of single compounds as well as extracts from plants and marine algae [11,12,13].

**Figure 1:**
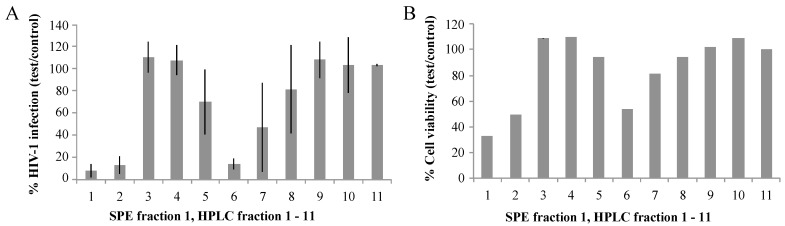
(**A**) HIV infection of RIC cells: Three biological replicates of 11 HPLC fractions (approx. 20 μg/mL each) generated from *S. carteri* SPE F1 were tested for the ability to inhibit HIV-1 replication in the EASY-HIT assay; (**B**) the cell viability of the infected LC5-RIC reporter cells treated with the three biological replicates of HPLC fraction 1–11 generated from *S. carteri* SPE F1 were assessed by the Microculture Tetrazolium Test (MTT). The error bars represent one standard deviation from the mean. *p* values from unpaired *t*-tests between the neighboring HPLC fractions are as follows for % infection and % cell viability: *p* = 0.43, 0.09 (HPLC 1:2), *p* = 0.0005, 0.0004 (HPLC 2:3), *p* = 0.8289, 0.8790 (HPLC 3:4), *p* = 0.1125, 0.1500 (HPLC 4:5), *p* = 0.0315, 0.4033 (HPLC 5:6), *p* = 0.2341, 0.0209, (HPLC 6:7), *p* = 0.3528, 0.3941 (HPLC 7:8), *p* = 0.3464, 0.5351 (HPLC 8:9), *p* = 0.7839, 0.0413 (HPLC 9:10), *p* = 0.9877, 0.0892(HPLC 10:11).

While all SPE fractions showed biological activities in the EASY-HIT assay, it was not possible to discriminate between effects on HIV infection and cell viability at this stage. Therefore, we exemplarily separated SPE fraction 1 (SPE F1) by HPLC into 11 separate fractions and evaluated their biological activities by EASY-HIT technology. As shown in Figure 1A, HPLC fractions 1, 2, and 6 strongly diminished expression of the HIV-reporter gene in the reporter cells, while the other HPLC fractions showed no or weaker inhibitory effects on the HIV infection parameter. HPLC fractions 2 and 6 also showed moderate effects on cell viability. Effects of HPLC fraction 1 on cell viability were stronger, resulting in elimination of HPLC fraction 1 for further analysis.

### 2.2. Identification of HIV-1 Candidate Inhibitors in HPLC Fractions 2 and 6

**Figure 2:**
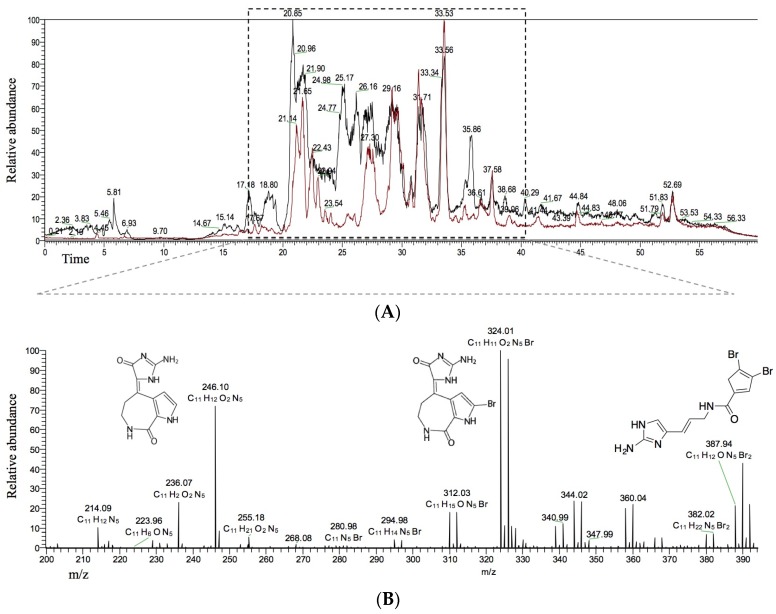
(**A**) overlay of LC-MS chromatograms for HPLC fractions 2 (**black**) and 6 (**red**) generated from *S. carteri* SPE fraction 1; and (**B**) a spectrum of the *m*/*z* values common to both HPLC fraction 2 and 6 between minutes 17–40. The three known compounds debromohymenialdisine (*m*/*z* [M + H]^+^ 246, C_11_H_12_N_5_O_2_), 10*Z*-hymenialdisine (*m*/*z* [M + H]^+^ 324, C_11_H_11_BrN_5_O_2_), oroidin (*m*/*z* [M + H]^+^ 389, C_11_H_12_Br_2_N_5_O) have the greatest relative abundance among the shared compounds common to both HPLC fractions 2 and 6.

To further evaluate potential HIV-inhibitory activity of *S. carteri*, we used LC-MS analysis to determine compounds common to HPLC fractions 2 and 6 as the most likely candidates for antiviral compounds (Figure 2A). The most prominent compounds common to both spectra included: debromohymenialdisine (DBH, *m*/*z* [M + H]^+^ 246, C_11_H_12_N_5_O_2_), hymenialdisine (HD, 10*Z*-hymenialdisine (*m*/*z* [M + H]^+^ 324, C_11_H_11_BrN_5_O_2_), and oroidin (*m/z* [M + H]^+^ 389, C_11_H_12_Br_2_N_5_O) (Figure 2B).

### 2.3. Three HIV-1 Candidate Inhibitors Hymenialdisine, Debromohymenialdisine, and Oroidin from S. carteri Inhibit HIV-1 Replication to Varying Degrees

**Figure 3:**
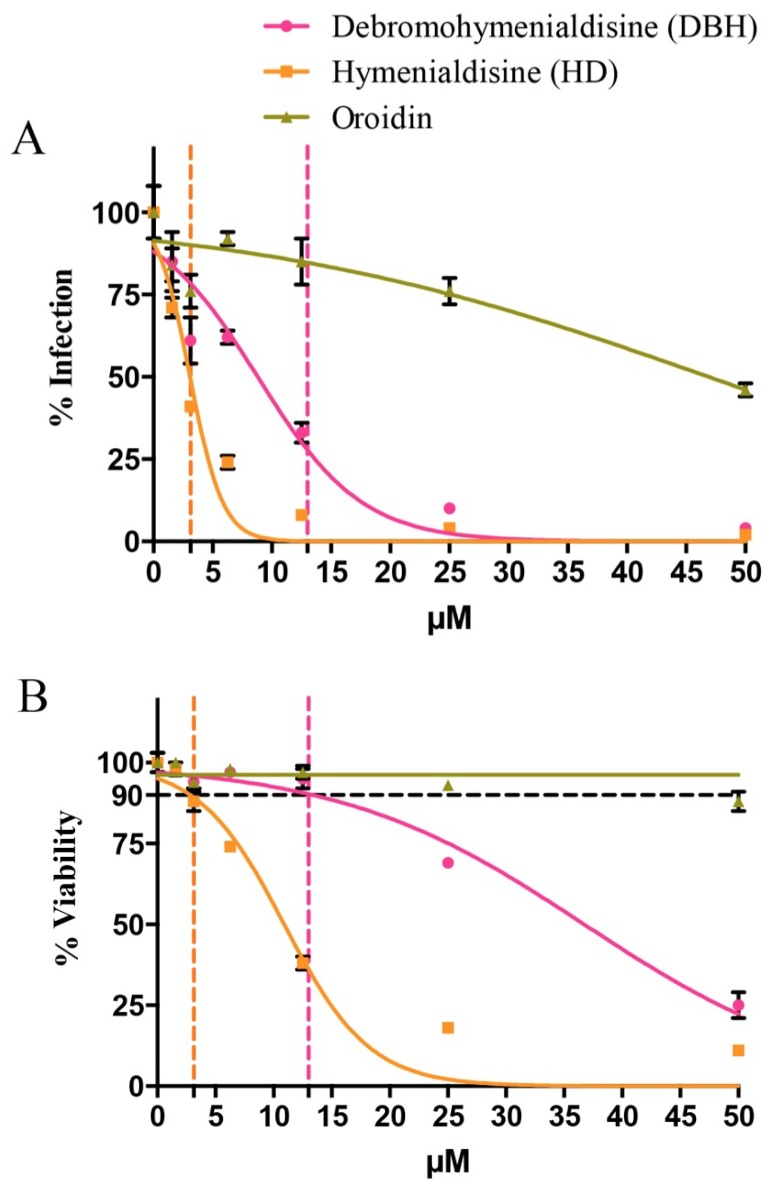
(**A**) the single compounds DBH, HD, and oroidin from *S. carteri* are tested for the ability to inhibit HIV-1 replication in HIV-1 permissible LC5-RIC reporter cells in the EASY-HIT assay; (**B**) cell viability of the infected LC5-RIC reporter cells treated with the single compounds DBH, HD, and oroidin from *S. carteri* was assessed by the MTT assay. The error bars represent one standard deviation from the mean. The *p* values between the six tested concentrations of each of the three compounds are reported first for the % infection and then % viability graph as follows: debromohymenialdisine *p* = 0.0001, *p* = 0.0001; hymenialdisine, *p* = 0.0001, *p* = 0.0001; oroidin, *p* = 0.0001, *p* = 0.0001.

Oroidin [14], HD and DBH [15,16], isolated and characterized in 1973 and the 1980s, respectively, have since been synthesized and are available for purchase. The single compounds were purchased and evaluated in the EASY-HIT assay for their ability to inhibit HIV-1 replication. The pure compounds were also used as standards and their presence was confirmed in the SPE F1 from the Red Sea *S. carteri* specimen collected in this study. Tandem mass spectrometry showed a similar retention time and fragmentation pattern for the commercial DBH, HD, and oroidin compounds and their corresponding compounds present in *S. carteri* SPE F1 (Figures S1 and S2). The DHB treatment inhibited HIV-1 infection down to 30% of untreated controls at a concentration of 13 μM without affecting cell viability (Figure 3A,B). The specific anti-viral effect was even stronger for HD, which clearly inhibited HIV infection to <40% at a concentration of 3.1 µM without affecting cell viability. Oroidin showed the weakest antiviral activity, reducing HIV-1 infection to 50% at a concentration of 50 μM, and proved to be nontoxic for cells at all tested concentrations (Figure 3A,B).

### 2.4. Oroidin from S. carteri Inhibits the HIV-1 Reverse Transcriptase (RT) in a Biochemical Assay

**Figure 4:**
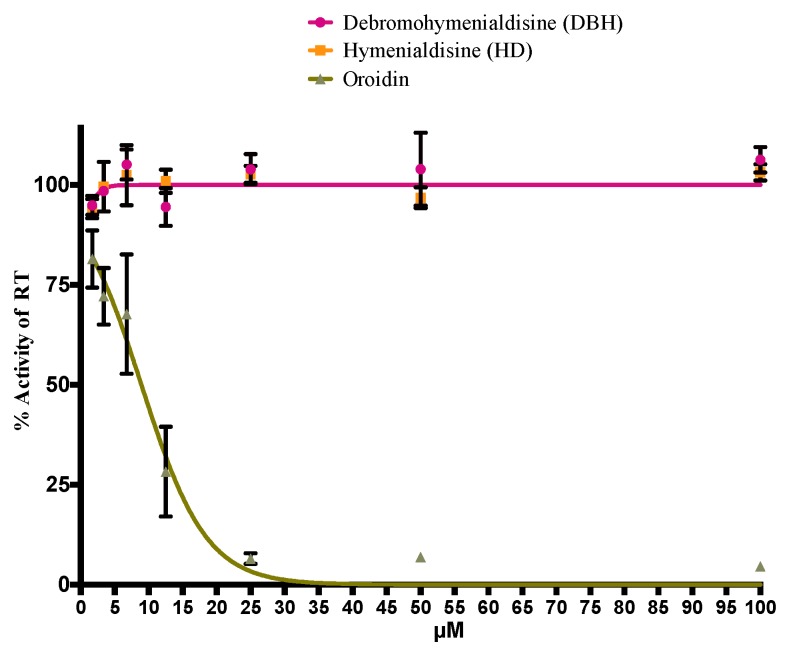
Inhibitory activity of DBH, HD and oroidin from *S. carteri* on the HIV-1 RT in a biochemical assay. The error bars represent one standard deviation from the mean. The *p* values are reported for % activity of the RT as follows: debromohymenialdisine, *p* = 0.0023; hymenialdisine, *p* = 0.169; oroidin, *p* = 0.0001.

The procured purified preparations of DBH, HD, and oroidin were screened in an HIV-1 RT biochemical assay. Interestingly, DBH and HD did not show anti-HIV-1 RT activity. In contrast, oroidin was capable of a 90% inhibition of the HIV-1 RT enzyme at concentrations greater than 25 μM (Figure 4). In the cell-based assay used here, oroidin was shown to reduce HIV-1 replication up to 50% when applied at a 50 μM concentration and is non-cytotoxic at all tested concentrations. This suggests that the mechanism of inhibition of oroidin as an HIV-1 RT inhibitor might be hindered by its poor ability to be absorbed by a living cell. Thus, improvements in oroidin as a prospective scaffold for HIV-1 inhibition would require additional medical chemistry modification. Our findings are in contrast to a previous study where oroidin was obtained as a by-product and was tested for its ability to inhibit HIV replication. In that study, oroidin was found inactive but also non-cytotoxic. The previous study [17] did not detail the concentrations used in the assay; however, their negative result may have been due to testing at too low a concentration of oroidin to observe activity in a cell-based assay.

Many of the pharmacologically active brominated pyrrole-2-aminoimidazole alkaloids from *S. carteri* are assumed to be derivatives of the building block oroidin [18]. In this study, oroidin was found to be capable of inhibiting HIV-1 replication without causing cytotoxicity. HD, a derivative of oroidin, was also found to be capable of inhibiting HIV-1 replication; however, cytotoxicity was observed at concentrations greater than 3.1 μM. This could be due to the ability of HD to bind Nuclear Factor-κB (NFκB), which is crucial for a number of cellular processes as well as the ability to induce the process of transcription from the HIV-1 long terminal repeat (LTR)*.* Similarly, DBH gives rise to cytotoxicity at concentrations greater than 13 μM, a value greater than that observed for HD, perhaps on account of its lack of the electronegative bromine atom. Furthermore, HD along with DBH have been reported to compete with ATP for binding to a number of cyclin-dependent kinases (CDK), including CDK2 [19], where the ability to inhibit CDK2 has been shown to decrease the transcription of the HIV-1 virus [18,20]. Additionally, DBH and HD have been reported to abrogate the G2-checkpoint of the cell cycle [21]. A G2 arrest is thought to pose a replicative advantage for the virus, as the HIV-1 LTR is most active during this phase [22]. This suggests that DBH and HD may also be able to relieve the cell cycle arrest that supports HIV pathogenesis; however, a prolonged arrest can also lead to apoptosis and result in cytotoxicity. NFκB, CDK2, and G2-checkpoint interference are three possible mechanisms of action to explain the inhibition observed from HD and DBH in this study; however, the inhibition of such ubiquitous cellular proteins and processes could also explain the cytotoxicity associated with HD and DBH as well as that observed for the parent SPE F1 mixture and in HPLC fractions 1, 2 and 6. From this study, it was observed that oroidin is directly acting to inhibit the retroviral Reverse Transcriptase, while DBH and HD do not affect this enzyme. It remains to be determined whether DBH and HD possess anti-viral activity at other steps of the virus life cycle that were not measured in this study. Similarly, future work should assess whether oroidin only affects the reverse transcriptase or whether other enzymes or life cycle steps are affected.

## 3. Experimental Section

### 3.1. S. carteri Specimen Collection and Sample Fractionation

Three biological replicates of *S. carteri* (described by Denny 1889, identified by Dr. N.J. de Voogd) were collected using gardening shears from three coral reef locations: C26 from Inner Fsar, West (22°13.974N, 39°01.760E), C14 from Inner Fsar, East (22°13.850N, 39°02.216E), and D5 from Al Fahal, East (22°15.119N, 38°57.761E), using SCUBA at approximately 12 m depth. The specimens were washed with 1% Phosphate Buffered Saline (PBS) on the boat then wrapped in foil and placed on ice then stored at −80 °C until processing. Small sponge specimens of 4–10 g of sponge specimen were ground using a mortar and pestle and then extracted with 15 mL of methanol overnight at 4 °C. The following day, the methanol extract was dried onto 150 mg of Diaion HP20SS beads in a CentriVap complete vacuum concentrator (Labconco, Kansas City, MO, USA) on low and the beads were then loaded into a 25 mL Flash Cartridge (Sorbtech, Norcross, GA, USA) with 0.795 diameter Frits (Sorbtech), desalted with deionized water (15 mL, FW1, FW2) and then eluted with 15 mL of 25% IPA/H_2_O (SPE fraction 1) [23]. The solvents was centrifugally evaporated in a CentriVap complete vacuum concentrator (Labconco, Kansas City, MO, USA) and re-dissolved in 2 mL HPLC grade methanol (Sigma Aldrich, Saint Louis, MO, USA) for injection into the HPLC.

#### 3.1.1. Analytical Chemistry of Bioactive *S. carteri* Fractions

The HPLC fractionation was peak-based and was carried out with a 50 μL (approximately 500 μg of material, dry weight) injection of material and a flow rate of 0.400 mL/min using a ZORBAX Eclipse XDB-C18 LC Column, 4.6 mm, 150 mm, 5 µm (Agilent Technologies, Santa Clara, CA, USA), and the gradient reported in Table S1. SPE fraction 1 and HPLC fractions 2 and 6 were analyzed on the Thermo LTQ Orbitrap with 10 µL of material and a flow rate of 0.800 mL/min using a ZORBAX Eclipse XDB-C18 LC Column, 4.6 mm, 150 mm, 5 µm and the gradient reported in Table S2. Analysis and prediction of elemental formulas were carried out with Xcalibur 2.1 software (Thermo Scientific, Waltham, MA, USA). MS/MS was preformed using a collision-induced dissociation (CID) of 30 eV on both the parent SPE fraction 1 as well as the three standards of DBH, HD and oroidin and targeted *m*/*z* 246.09, 324.00, and 389.93, respectively.

#### 3.1.2. Single Compounds from Bioactive *S. carteri* Fractions

The individual synthesized compounds debromohymenialdisine (Lot: L20314, Enzo Life Sciences, Farmindale, NY, USA), 10*Z*-hymenialdisine (Lot: L27315, Enzo Life Sciences) and oroidin (Lot: L26427, Enzo Life Sciences) (Figure S3) were ordered screened on the EASY-HIT assay and the HIV-1 RT biochemical assay at molarities below 100 μM in order to assess their ability inhibit HIV-1 replication. Statistical significances were determined using one-way ANOVA.

### 3.2. Human Immunodeficiency Virus, Full Virus Screening (EASY-HIT)

The EASY-HIT assay [11] is based on HIV-1 susceptible reporter cells (LC5-RIC) that contain a stably integrated fluorescent reporter gene that is activated upon successful HIV-1 infection and expression of the early viral proteins Rev and Tat. LC5-RIC were seeded into 96-well plates (μCLEAR-Plate Black; Greiner Bio-One, Kremsmuenster, Germany) using only the 60 inner wells to avoid variations in the culture conditions in the outer wells. Cells were seeded at a density of 10,000 cells per well 24 h prior to infection. SPE fractions for the biological replicates C26, C14 and D5, were tested in a serial 1:2-dilution with a maximum of 3% methanol or approximately 75 μg/mL for the *S. carteri* fraction 1 mixture on the EASY-HIT assay [11]. For the HPLC fractions, SPE fraction 1 HPLC fractions 1–11, were tested on the EASY-HIT assay for biological replicates C26, C14 and D5. The authentic samples of HD, DBH, and oroidin were screened at 3.1 μM, 6.25 μM, 12.5 μM, 25 μM, and 50 μM. All samples were tested in triplicate. After compound addition, LC5-RIC cells were infected by adding 20 μL of HIV-1 inoculum (approx. 28.8 ng of p24 for HIV-1LAI derived from HEK 293T cells) to each well of the plate. Cells were incubated at standard cell culture conditions for 48 h after infection and were subsequently assayed for reporter expression and cell viability. Reporter expression was determined by measuring the total fluorescent signal intensity of each culture with a fluorescence microplate reader (Fluoroskan Ascent; ThermoFisher, Schwerte, Germany) at an excitation filter wavelength of 544 nm and an emission filter wavelength of 590 nm or with a Tecan infinite M200 (Tecan, Crailsheim, Germany) at the monochromator wavelengths 552 nm for excitation and 596 for emission. Statistical significances were determined using unpaired *t*-tests for neighboring HPLC fractions.

#### MTT Cell Proliferation Assay

The colorimetric assay MTT was used in order to assess the viability and activity of LC5-RIC cells exposed to the unknown sponge mixtures, SPE fraction 1 and HPLC fractions 1–11, for three biological replicates. This cell viability assay provides a visualization of the process where mitochondrial enzymes reduce the yellow MTT to purple formazan (American Type Culture Collection (ATCC), MTT cell proliferation assay). After reading the HIV reporter expression, cultures were incubated with 100 μL of MTT solution (0.5 mg of MTT; Sigma, Taufkirchen, Germany) in 100 μL of culture medium for 2 h. MTT solution was removed and 100 μL of lysis solution (10% (*w/v*) SDS and 0.6% (*v/v*) acetic acid in dimethyl sulfoxide (DMSO)) was added. The formazan concentrations of the test compounds and the uninfected control cultures were determined by an ELISA plate reader (Tecan Infinite M200, Tecan Germany GmbH, Crailsheim, Germany) and scanned with a test wavelength of 570 nm and a reference wavelength of 630 nm. Statistical significances were determined using one-way ANOVA.

### 3.3. Human Immunodeficiency Virus, Reverse Transcriptase Biochemical Test

The HIV-1 reverse transcriptase inhibition assay was carried out using the commercial kit EnzChek^®^ Reverse Transcriptase Assay (Invitrogen, San Jose, CA, USA). The reverse transcriptase is a heterodimer with the p66 and the p51 subunits with RNase H activity located in the last 15KDa of the p66 HIV Reverse transcriptase (Calbiochem, Merck-Millipore, Billerica, MA, USA). Polymerase activity was assessed by its ability to produce RNA-DNA heteroduplexes from a mixture of a long poly (A) template, an oligo dT primer and dTTP which is then detected by PicoGreen^®^ dsDNA quantitation reagent. The single compound and controls were performed in triplicated in a 384-well plate format, each with a total reaction mixture of 50 μL. To begin, the poly (A) template was annealed to the oligo dT primer for one hour, then diluted 200 fold in polymerization buffer. Reverse transcriptase (1.2 μL) was added per 300 reactions. This mixture was kept on ice and aliquoted to each well with test compounds. The reaction was incubated at room temperature for one hour and then 2 μL of 50 nM EDTA was added to stop the reaction. The RNA-DNA heteroduplex was labeled with PicoGreen and incubated for 5 min and then detected with the use of a SpectraMax^®^ Paradigm^®^ Multi-mode Microplate Detection Platform (Molecular Devices, Sunnyvale, CA, USA) by scanning at 480 nm excitation wavelength and 520 nm emission wavelength. The authentic standards of HD, DBH, and oroidin were screened at 3.1 μM, 6.25 μM, 12.5 μM, 25 μM, 50 μM, and 100 μM. Statistical significances were determined using a one-way ANOVA.

## 4. Conclusions

*S. carteri* is a sponge with wide geographical distribution that has been shown to produce bioactive secondary metabolites, some of which are also present in members of three other sponge families. The three metabolites detected in this study, DBH, HD and oroidin, were characterized 30–40 years ago. This investigation revealed that these compounds are able to inhibit HIV-1 replication, and could serve as starting scaffolds for further investigation. The long time lag between the original discovery of these compounds and this report on their anti-retroviral activity accentuates the unevenness of the screening efforts of natural products and the need for diversification in assay targets. The broader implication of this observation is that the anti-viral pharmacophore potential of many marine natural products might currently be overlooked. In the future, anti-viral screening efforts similar to this one could reveal new marine-derived agents useful in combating these devastating viral diseases.

## Supplementary Files

Supplementary File 1
