# Supplementary material for: Alkaloids from the Sponge Stylissa carteri Present Prospective Scaffolds for the Inhibition of Human Immunodeficiency Virus 1 (HIV-1)
Source: Mar Drugs. 2016 Feb 4;14(2):28. doi: 10.3390/md14020028 (PMC4771981; doi:10.3390/md14020028)
Supplement: Supplementary File 1 [file marinedrugs-14-00028-s001.pdf]

Supplementary Materials: Alkaloids from the Sponge *Stylissa carteri* Present Prospective Scaffolds for the Inhibition of Human Immunodeficiency Virus 1 (HIV-1)

Aubrie O'Rourke, Stephan Kremb, Theresa Maria Bader, Markus Helfer, Philippe Schmitt-Kopplin , William H. Gerwick, Ruth Brack-Werner and Christian R. Voolstra

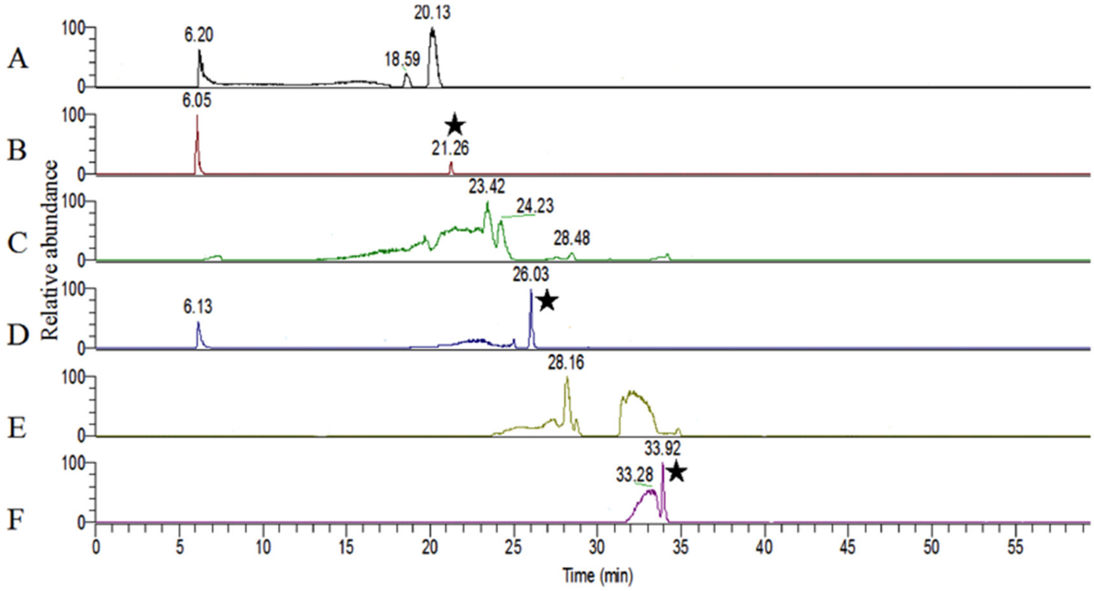

Figure S1. LC-MS chromatograms (exctracted ion chromatograms) of the *m*/*z* ions associated with debromohymenialdisine, hymenialdisine, and oroidin for SPE fraction 1 from *S. carteri* and comparison to the purchased standards. (**A**) retention time for debromohymenialdisine in the SPE fraction 1 of *S. carteri*; (**B**) retention time for the debromohymenialdisine standard (starred); (**C**) retention time for hymenialdisine in the SPE fraction 1 of *S. carteri*; (**D**) retention time for the hymenialdisine standard (starred); (**E**) retention time for oroidin in the SPE fraction 1 of *S. carteri*; (**F**) retention time for the oroidin standard (starred).

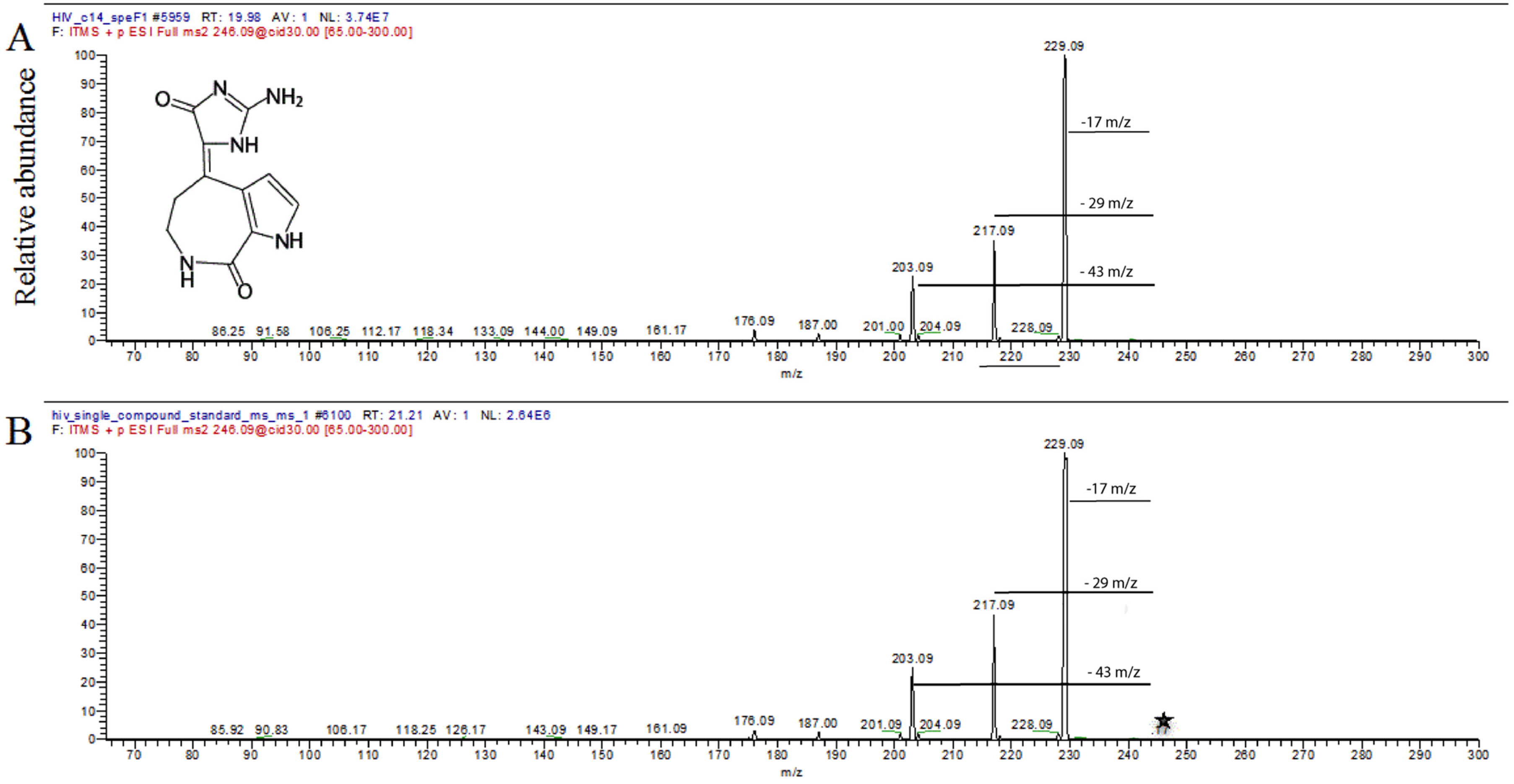

Figure S2. *Cont.*

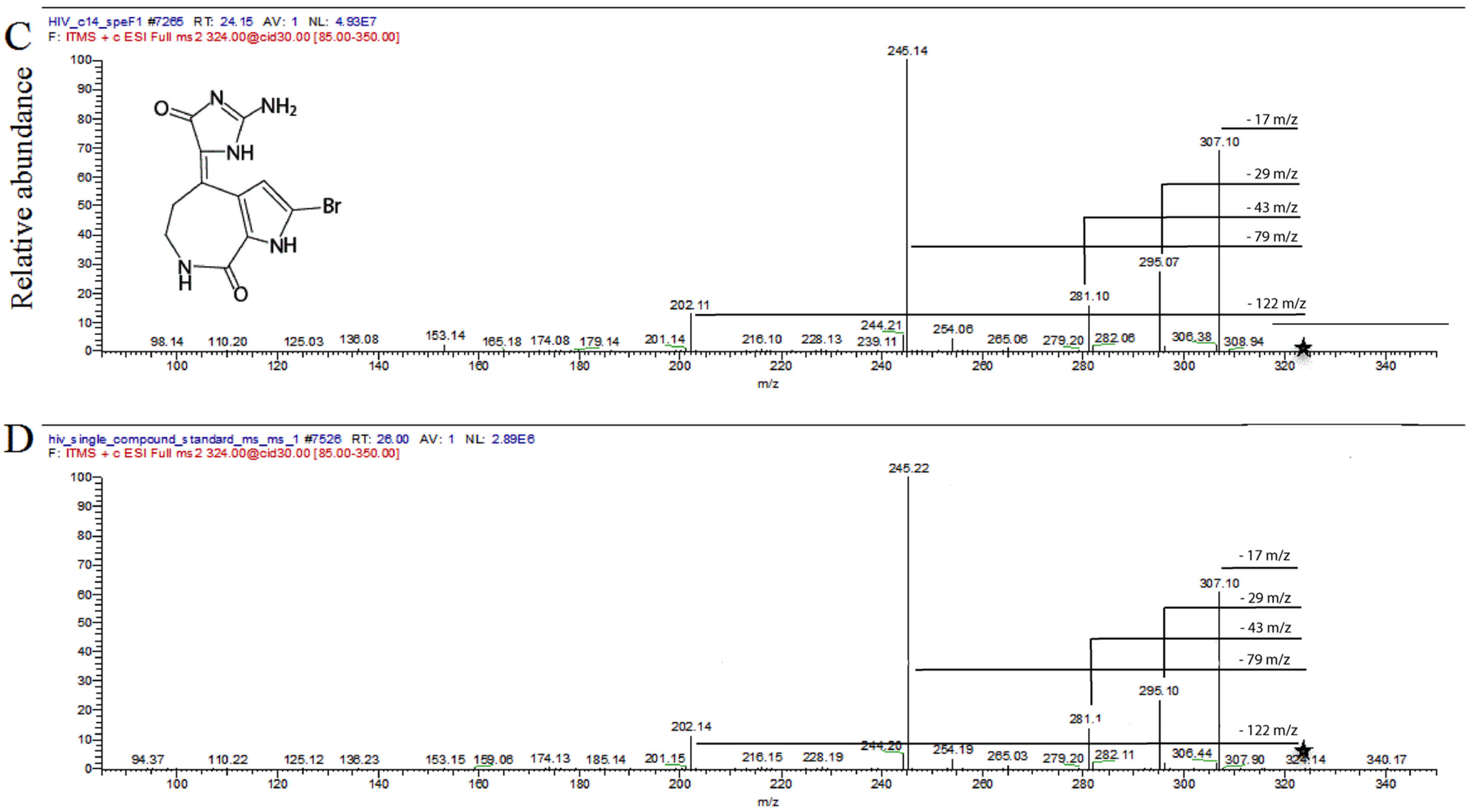

Figure S2. *Cont.*

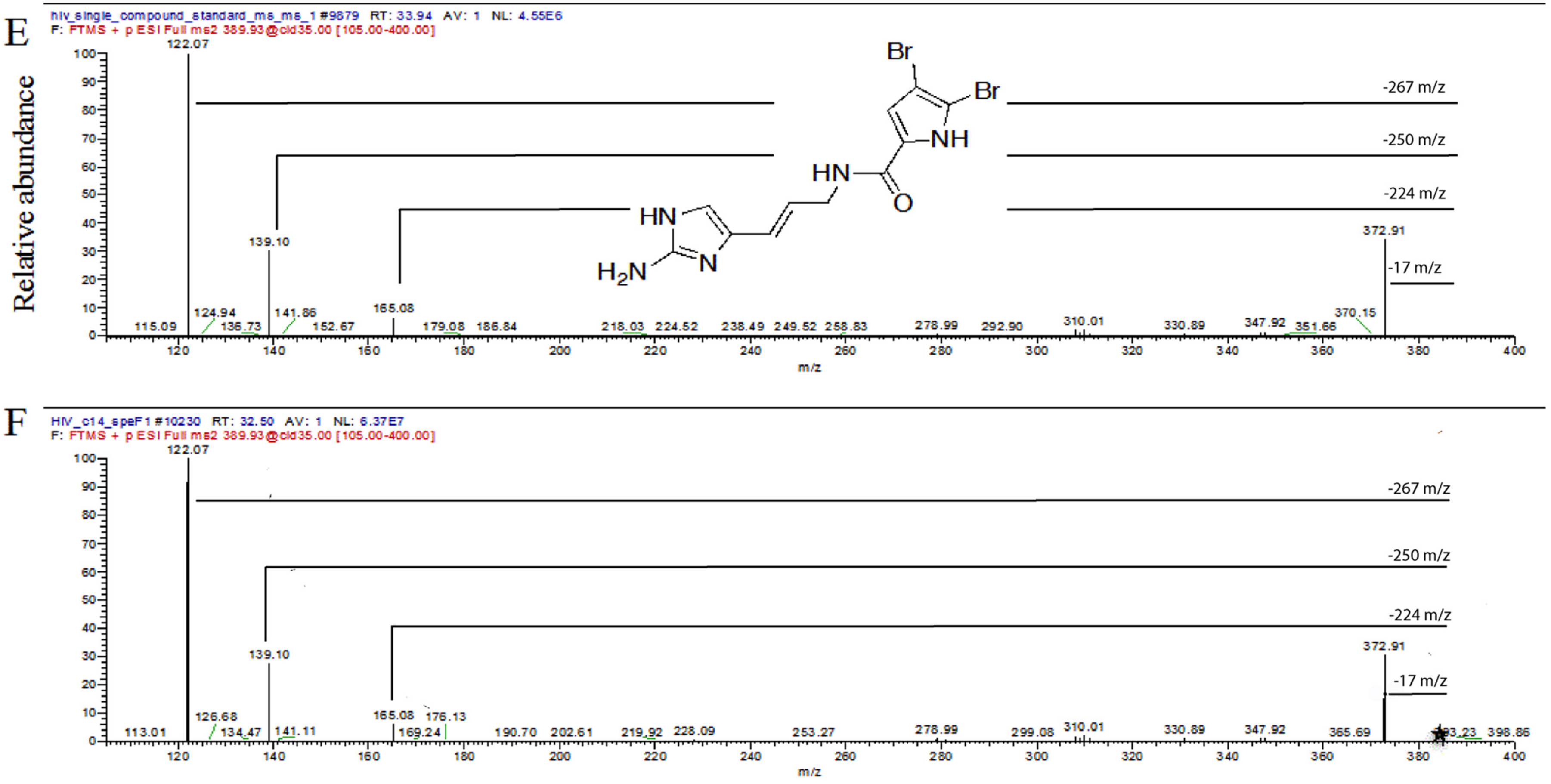

Figure S2. MS/MS fragmentation pattern for debromohymenialdisine, hymenialdisine, and oroidin using a collision-induced dissociation of 30 eV. (**A**) Fragmentation pattern of debromohymenialdisine in the SPE fraction 1 of *S. carteri*;(**B**) Fragmentation pattern of the debromohymenialdisine standard; (**C**) Fragmentation pattern of hymenialdisine in the SPE fraction 1 of *S. carteri*; (**D**) Fragmentation pattern of the hymenialdisine standard; (**E**) Fragmentation pattern of oroidin in the SPE fraction 1 of *S. carteri*; (**F**) Fragmentation pattern of the oroidin standard.

Debromohymenialdisine

Product Specifications

Alternative Name:	DBH, 4-(2-Amino-4-oxo-2-imidazolin-5-ylidene)-4,5,6,7-tetrahydropyrrolo[2,3-*c*]azepin-8-one
IDENTITY:	Identity determined by ^{1}H-NMR and MS.
FORMULA:	$C_{11}H_{11}N_5O_2$
MW:	245.2
PURITY:	≥95% (HPLC)
APPEARANCE:	Yellow solid.
CAS:	75593-17-8, 125118-55-0
SOURCE/HOST:	Isolated from sponge *Axinella carteri*.
SOLUBILITY:	Soluble in 100% ethanol or DMSO.
LONG TERM STORAGE:	-20°C
HANDLING:	Keep under inert gas.<br>Protect from light.

Hymenialdisine

Product Specifications

IDENTITY:	Identity determined by ^{1}H- NMR and MS.
FORMULA:	$C_{11}H_{10}BrN_5O_2$
MW:	324.1
PURITY:	≥97% (HPLC)
APPEARANCE:	Yellow oil.
CAS:	82005-12-7
SOURCE/HOST:	Isolated from sponge *Axinella carteri*.
SOLUBILITY:	Soluble in DMSO (5mg/ml).
SHIPPING:	AMBIENT
LONG TERM STORAGE:	-20°C
HAZARD:	TOXIC.
HANDLING:	Protect from light.

Oroidin

Product Specifications

FORMULA:	$C_{11}H_{11}Br_2N_5O$
MW:	389.1
CAS NUMBER:	34649-22-4
SOURCE/HOST:	Isolated from S*tylissa* sp.
PURITY:	≥97% (HPLC, NMR)
IDENTITY:	Identity determined by ^{1}H-NMR, ^{13}C-NMR and MS.
APPEARANCE:	Amorphous solid. (Actual lot: beige solid)
SOLUBILITY:	Soluble in 100% ethanol or DMSO.
SHIPPING:	AMBIENT
LONG TERM STORAGE:	-20°C
HANDLING:	Protect from light. Keep cool and dry.

Figure S3. Enzo Life Sciences quality control reports for the procured debromohymenialdisine (Lot: L20314), hymenialdisine (Lot: L27315), and oroidin (Lot: L26427).

Table S1. HPLC gradient used to separate the *S. carteri* SPE fraction 1 into HPLC fractions 1–11.

Time	% H_2O	% Acetonitrile
0 min	65	35
1 min	65	35
20 min	30	70
26 min	30	70
28 min	65	35
30 min	65	35

Table S2. LC-MS gradient used to characterize the bioactive compounds of *S. carteri* SPE fraction 1, HPLC fraction 2 and 6.

Time	% H_2O	% MeOH	Formic Acid in Each Solvent
0 min	90	10	0.10%
5 min	90	10	0.10%
40 min	10	90	0.10%
50min	10	90	0.10%
55min	90	10	0.10%
60min	90	10	0.10%
